# Precise stellarator quasi-symmetry can be achieved with electromagnetic coils

**DOI:** 10.1073/pnas.2202084119

**Published:** 2022-03-22

**Authors:** Florian Wechsung, Matt Landreman, Andrew Giuliani, Antoine Cerfon, Georg Stadler

**Affiliations:** ^a^Courant Institute of Mathematical Sciences, New York University, New York, NY 10012;; ^b^Institute for Research in Electronics and Applied Physics, University of Maryland, College Park, MD 20742

**Keywords:** fusion, quasi-symmetry, confinement, magnetic coils

## Abstract

Magnetic fields with quasi-symmetry are known to provide good confinement of charged particles and plasmas, but the extent to which quasi-symmetry can be achieved in practice has remained an open question. Recent work [M. Landreman and E. Paul, *Phys. Rev. Lett.* 128, 035001, 2022] reports the discovery of toroidal magnetic fields that are quasi-symmetric to orders-of-magnitude higher precision than previously known fields. We show that these fields can be accurately produced using electromagnetic coils of only moderate engineering complexity, that is, coils that have low curvature and that are sufficiently separated from each other. Our results demonstrate that these new quasi-symmetric fields are relevant for applications requiring the confinement of energetic charged particles for long time scales, such as nuclear fusion. The coils’ length plays an important role for how well the quasi-symmetric fields can be approximated. For the longest coil set considered and a mean field strength of 1 T, the departure from quasi-symmetry is of the order of Earth’s magnetic field. Additionally, we find that magnetic surfaces extend far outside the plasma boundary used by Landreman and Paul, providing confinement far from the core. Simulations confirm that the magnetic fields generated by the new coils confine particles with high kinetic energy substantially longer than previously known coil configurations. In particular, when scaled to a reactor, the best found configuration loses only 0.04% of energetic particles born at midradius when following guiding center trajectories for 200 ms.

Controlled nuclear fusion is a promising candidate to satisfy rising electricity needs while avoiding carbon emission into the atmosphere. To generate electricity from fusion, a plasma needs to be maintained at extremely high temperatures over long time scales, which requires excellent particle confinement. This is typically achieved using powerful magnets. Tokamaks rely on a toroidally axisymmetric system of magnetic coils to achieve good confinement, at the cost of requiring a plasma current to generate a significant fraction of the magnetic field. This plasma current is challenging to drive in steady-state operation (1), and can be the source of disruptive instabilities (2, 3). In contrast, the nonaxisymmetric coil systems of stellarators can generate a confining magnetic field in the absence of plasma currents, relieving many of the challenges for continuous, disruption-free operation. However, the lack of axisymmetry implies that neither nested magnetic flux surfaces nor particle confinement is guaranteed (4). This motivates the need for a generalization of axisymmetry to the stellarator context, called quasi-symmetry.

A magnetic field is said to satisfy quasi-symmetry if there exists an invariant direction for the field strength B=|B| in a certain coordinate system (5). This condition leads to the conservation of canonical angular momentum, which, in turn, implies the remarkable property that such fields are guaranteed to confine charged particles without requiring plasma currents. However, it remains an open question whether three-dimensional magnetic fields that are perfectly quasi-symmetric over a volumetric region exist. Recently, using numerical optimization, Landreman and Paul (6) found vacuum magnetic fields that satisfy the quasi-symmetry property in toroidal geometries to a very high precision. Magnetic fields that are quasi-symmetric to such a high degree have not been discovered before. Simulations confirm excellent confinement properties even for particles with a large kinetic energy. Naturally, the question arises whether these magnetic fields can be accurately produced by a set of practical electromagnets, which would be a first step toward a new generation of fusion experiments. In this report, we show that such magnets, in fact, exist. Specifically, we find coils producing fields whose deviation from perfect quasi-symmetry is more than four orders of magnitude smaller than the mean field strength. For a 1-T mean field, comparable to many stellarator experiments, this error can be on the order of Earth’s magnetic field. Simulations confirm particle confinement comparable to the ideal fields discovered in ref. 6. This shifts the discovery of these highly quasi-symmetric fields from one of theoretical interest to one of practical relevance for fusion experiments and other confinement applications (7).

## Results and Discussion

We solve a constrained optimization problem to obtain coils that approximate the two quasi-axisymmetric fields of ref. 6, which we refer to as “QA-LP” and “QA+Well-LP,” reflecting the absence or presence of a magnetic well. The design space consists of four distinct modular coils, which results in 16 coils in total after applying symmetries. The length of coils impacts the quality of the magnetic field approximation strongly, and hence we compute coil sets of different coil length $L_{\max}$ and compare their performance, for example, “QA+Well[20]” refers to the coil set which approximates the “QA+Well-LP” configuration and for which the four modular coils have combined length 20 m. For both “QA-LP” and “QA+Well-LP,” as we allow longer coils, the field induced by the coils becomes a better approximation of the target field. Values of $L_{\max}$ are relative to an average major radius of 1 m.

**Fig. 1.**
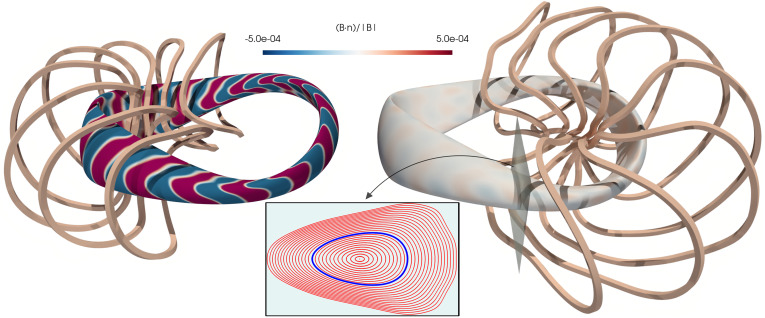
Half of the coils for the QA[18] (*Left*) and QA[24] (*Right*) configurations and resulting relative normal component of the magnetic field on the target surface. The full set of 16 coils satisfies twofold rotational symmetry. (*Insert*) Poincaré plot for the QA[24] configuration, obtained by numerically tracing magnetic field lines and recording their intersection with the shown cross-section. Good flux surfaces extend far outside the boundary from ref. 6 (highlighted in blue). We note that the rectangular cross-section of the coils is for visualization only; in all computations for this manuscript, the coils are each approximated by a single wire of infinitesimal thickness. We conducted numerical experiments in which we approximated finite-thickness coils using multiple filaments, and obtained very similar results, not shown here.

Fig. 1 shows coils obtained by choosing $L_{\max}=18\;\text{m}$ and $L_{\max}=24\;\text{m}$ in the approximation to QA-LP, the relative normal magnetic field B·n/|B| on the surface *S*, and a Poincaré plot for $L_{\max}=24\;\text{m}$. Here **B** is computed using the Biot–Savart law, and, if the normal magnetic field is exactly zero, then the field matches that discovered in ref. 6 everywhere in the volume contained by the surface. For the shortest coils, we observe the largest normal magnetic field on the surface, reaching values of up to $3.3\times 10^{-3}$. Its oscillatory nature is caused by the discrete nature of the electromagnetic coils and their close proximity to the surface. Longer coils enable a larger distance between the surface and the magnets, thereby reducing these discrete effects, and more accurately reproducing the target magnetic field, with a relative normal magnetic field of, at most, $1.6\times 10^{-4}$ for the QA[24] configuration.

The Poincaré plot demonstrates the existence of nested magnetic surfaces in the entire volume. This feature is necessary (but not sufficient) for confinement, since charged particle trajectories are tangent to these surfaces in the limit of low energy.

**Fig. 2.**
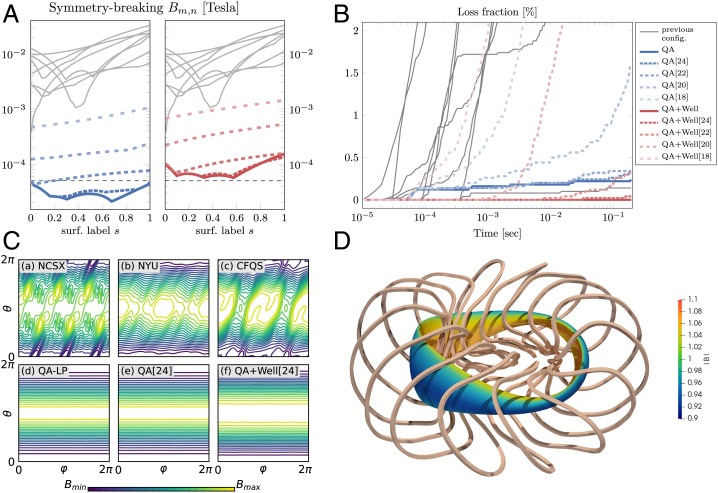
(*A*) Magnitude of the largest symmetry-breaking Fourier modes of |B| for surfaces with normalized toroidal flux *s* (*s* = 1 corresponds to the target surface). All configurations are scaled to a mean field strength of 1 T. The dashed line indicates 50 µ T, which is approximately the strength of Earth’s magnetic field. (*B*) Losses of alpha particles spawned on the *s* = 0.25 surface. Apart from the Wistell-A configuration, all previous configurations lose 10% or more of energetic particles within 0.2 s. The QA+Well[24] configuration gets extremely close to the perfect confinement of the QA+Well-LP configuration, with only 0.04% particles being lost. (*C*) Comparison between magnetic field strength on the boundary surface of previous magnetic field configurations designed to approximate quasi-symmetry (*a*–*c*), and the precise quasi-symmetric designs with coils presented in this paper (*d–f*). Shown in *a* is the National Compact Stellarator Experiment (NCSX), *b* is a QA developed at New York University (NYU) (8), and *c* is the Chinese First Quasiaxisymmetric Stellarator (CFQS). Shown in *d* are the field lines from the design without coils from ref. 6, and, in *e* and *f*, those obtained by using coils of length 24 to approximate the QA-LP and the QA+Well-LP configurations. (*D*) The coils corresponding to the QA+Well[24] configuration. The color indicates the magnetic field strength |B|.

Quasi-axisymmetric fields are characterized by the following property: When parametrized using Boozer coordinates ϕ,θ, the field strength on each magnetic surface only depends on the angle *θ* (5, 8). When this property is satisfied exactly, even highly energetic collisionless particles are confined over long time scales, and collisional transport is minimized. In Fig. 2*C*, we compare the magnetic field strengths of three earlier stellarator configurations with the QA-LP field as well as our QA[24] and QA+Well[24] fields. The QA[24] field strength is visually indistinguishable from that of the QA-LP configuration; the same holds true for QA+Well-LP and QA+Well[24].

We quantify this statement by performing a Fourier transformation of |B| and then studying the magnitude of those Fourier coefficients that break quasi-symmetry; that is, we write $\left|B(s,\theta ,\phi )|=\sum_{m,n}B_{m,n}(s)\cos\;(m\theta -n\phi \right)$ and then consider terms with n≠0. Here *s* indexes each surface by the normalized toroidal magnetic flux it encloses, and we plot the largest symmetry-breaking Fourier mode on each surface in Fig. 2*A*. As coil length is increased, the symmetry-breaking error is reduced significantly, and approximates those of the fields discovered in ref. [6]. In fact, we are able to achieve errors smaller than Earth’s magnetic field for the longest set of coils when the mean field strength is 1 T. For comparison, gray curves show the symmetry-breaking amplitudes for the eight previous quasi-symmetric configurations in figure 1 of ref. 6.

Finally, scaling the configurations to the mean field and minor radius of the ARIES-CS reactor (9), we compute collisionless guiding center trajectories for alpha particles initialized with a kinetic energy of 3.5 MeV, as by-products of a deuterium–tritium fusion reaction, on the surface with normalized toroidal flux *s* = 0.25 (half radius). Particle losses are shown in Fig. 2*B*. For $L_{\max}=24\;\text{m}$, the performance is nearly indistinguishable from the target equilibrium, and, for the QA+Well[24] configuration, less than 0.04% of particles are lost after 0.2 s, a typical time for the alphas to thermalize with the main plasma. This coil set is shown in Fig. 2*D*. Performance is only slightly worse for $L_{\max}=22\;\text{m}$, but is poor for the coils with $L_{\max}=18\;\text{m}$. For comparison, gray curves show calculations for the nine previous stellarator configurations of figure 6A in the SI Appendix of ref. 6, similarly scaled. We note that the thermal collisional transport magnitude $\epsilon_{\text{eff}}^{3/2}$ (10) is less than $2\times 10^{-7}$ for coil lengths of $L_{\max}=22\;\text{m}$ and $L_{\max}=24\;\text{m}$ for both the QA and the QA+Well configurations, and is $<6\times 10^{-8}$ for QA[24]. These values are orders of magnitude below the values for other optimized stellarators [e.g., $∼10^{-3}$ for Wendelstein 7-X (4)], and are so small that collisional fluxes would be negligible compared to turbulent transport. This is the standard situation for tokamaks, but is unusual and desirable for stellarators, since collisional and turbulent losses are additive (4).

In conclusion, we have shown that the magnetic fields of ref. 6 can be produced very accurately using coils, making these fields practically relevant for stellarators. As a result, exceptionally good confinement of particle trajectories and remarkably small thermal collisional transport can be achieved. While longer coils are required for optimal performance, these coils are not particularly complex as measured in terms of curvature and coil-to-coil separation.

## Materials and Methods

The electromagnetic coils were optimized using the SIMSOPT software (11). Similar to the approach in ref. 12, each coil is modeled as a closed, smooth curve in $R^{3}$ and represented using a Fourier series, truncated at order 16. Given a magnetic surface *S* (obtained from ref. 6), the objective that we minimize is given by

[1] $$f_{B}=\int_{S}{\left(\frac{B\cdot n}{\left|B\right|}\right)}^{2}\text{ds},$$

where **B** is the field induced by the coils. If *f_B_*  =  0, then the induced field is exactly equal to the target field up to a scaling factor.

Finding coils that minimize this objective is an ill-posed problem, so we require additional regularization. In practice, it is desirable to have coils that are not too long, avoid high curvature, and are well separated. In this work, we enforce constraints on the curvature ($\kappa_{\max}\leq 5\;{\text{m}}^{-1}$), the mean squared curvature ($\kappa_{\text{msc}}\leq 5\;{\text{m}}^{-2}$), and the distance between coils ($d_{\min}\geq 0.1\;\text{m}$), and we vary the constraint on the total length of the magnetic coils ($L_{\max}\in \{18,20,22,\text{and}\;24\;\text{m}\}$). The units quoted in the above assume a major radius scaled to 1 m. We refer to *SI Appendix* for more detail on the exact implementation of these constraints.

The optimization objective has 399 parameters for the coils and is highly nonconvex. The minimization uses the L-BFGS-B algorithm with analytic gradients. To remedy the possibility of the optimizer being stuck in a local minimum, we start the optimization from eight different initial coil sets and choose the best minimizer as measured by the objective. The code is highly optimized and parallelized and is publicly available at https://github.com/florianwechsung/CoilsForPreciseQS. Using eight cores of an Intel Xeon Platinum 8268 CPU, solving an individual optimization problem takes ∼30 min.

## Supplementary Material

Supplementary File

## Data Availability

Code and optimization results have been deposited in GitHub (https://github.com/florianwechsung/CoilsForPreciseQS) and Zenodo (10.5281/zenodo.5975323) (13).
