# Supplementary material for: Precise stellarator quasi-symmetry can be achieved with electromagnetic coils
Source: Proc Natl Acad Sci U S A. 2022 Mar 22;119(13):e2202084119. doi: 10.1073/pnas.2202084119 (PMC9060451; doi:10.1073/pnas.2202084119)
Supplement: Supplementary File [file pnas.2202084119.sapp.pdf]

Supplementary Information for

Precise stellarator quasi-symmetry can be achieved with electromagnetic coils

Florian Wechsung, Matt Landreman, Andrew Giuliani, Antoine Cerfon, Georg Stadler

Corresponding author: Florian Wechsung.
E-mail: wechsung@nyu.edu

This PDF file includes:

Supplementary text

Supporting Information Text

Supplementary Material: formulation of the optimization problem

For completeness and to aid reproducibility, we give additional detail on the formulation of the optimization problem that we solve in order to obtain the coils in this note. Coil i is described by a periodic function $\mathbf{\Gamma}^{(i)} : [0, 2\pi) \to \mathbb{R}^3$, where $\mathbf{\Gamma}^{(i)} = [\Gamma_1^{(i)}, \Gamma_2^{(i)}, \Gamma_3^{(i)}]$ and

$$\Gamma_j^{(i)}(\theta) = c_{j,0}^{(i)} + \sum_{l=1}^{N_F} s_{j,l}^{(i)} \sin(l\theta) + \sum_{l=1}^{N_F} c_{j,l}^{(i)} \cos(l\theta). \tag{1}$$

In this work we choose $N_F = 16$.

We consider four distinct coils and collect their degrees of freedom in a vector $\mathbf{c}$. By applying two-fold rotational symmetry as well as stellarator symmetry this results in 16 coils in total. The input to the optimization problem is a surface $S \subset \mathbb{R}^3$ and the goal is to find coils that induce a magnetic field that is tangential to the surface everywhere. This is achieved by considering the objective

$$f_B(\mathbf{c}) = \int_S \left(\frac{\mathbf{B}(\mathbf{c}) \cdot \mathbf{n}}{\|\mathbf{B}(\mathbf{c})\|} \right)^2 \mathrm{d}s. \tag{2}$$

In addition to this objective, we consider several constraints that restrict the geometry of the coils. That is we solve,

$$\begin{aligned} \text{minimize}_{\mathbf{c}} \quad & f_B(\mathbf{c}) \\ & L_1 + L_2 + L_3 + L_4 \leq L_{\max} \\ \text{subject to} \quad & \mathrm{Max}(\kappa_i) \leq \kappa_{\max} \text{ for all } i \\ & \mathrm{Mean}(\kappa_i^2) \leq \kappa_{\mathrm{msc}} \text{ for all } i \\ & \min_{\theta, \theta'} \|\Gamma_i(\theta) - \Gamma_j(\theta')\| \geq d_{\min} \text{ for all } i \neq j \\ & \ell_0^{(i)} = \ldots = \ell_{2N_F - 1}^{(i)} \text{ for all } i. \end{aligned} \tag{3}$$

Here L_i is the length and κ_i is the curvature of curve i. Apart from the last, these constraints are all similar to those used in previous coil design work, see e.g. (? ? ?). It remains to explain the quantity $\ell_j^{(i)}$. To motivate the need for an additional constraint, we note that the parametrization Eq. (1) is not unique, in the sense that given any smooth, strictly monotonic, bijective function $r : [0, 2\pi) \to [0, 2\pi)$, an equivalent curve is given by $\theta \mapsto \Gamma_j^{(i)}(r(\theta))$. To avoid poor conditioning of the optimization problem due to this nonuniqueness, we include a term in the objective that (weakly) enforces a uniform arclength along the curve. One way of doing this would be to penalize the variance of the incremental arclength $|\mathbf{\Gamma}'^{(i)}|$ along the curve. We relax this approach slightly by instead considering the average arclength on a number of intervals, and then penalize the value the variance of those values. The intuition behind this approach is given by the fundamental theorem of curves: the theorem states that any curve with nonzero curvature can be uniquely specified by an initial position and direction as well as its curvature, torsion, and arc length along the curve. Since each coil has $3(2N_F + 1)$ degrees of freedom, subtracting six degrees of freedom for the initial position and direction, this leaves $6N_F - 3$ degrees of freedom that represent curvature, torsion, and arclength. Hence we want to add $2N_F - 1$ constraints to the optimization problem, which corresponds to requiring that the variance of the arclength on $2N_F$ intervals should be zero. More explicitly, we define

$$\ell_j^{(i)} = ⨍_{[j/(2N_F), (j+1)/(2N_F)]} |\mathbf{\Gamma}'^{(i)}(\theta)| d\theta. \tag{4}$$

In order to enforce these constraints, we consider the following penalty functions

$$\begin{aligned} g_L(\mathbf{c}) &= \varphi\big([\textstyle\sum_{i=1}^{N_c} L_i] - L_{\max}\big) \\ g_{\kappa,\max}(\mathbf{c}) &= \sum_{i=1}^{N_c} \frac{1}{L_i} \int_0^{2\pi} \varphi(\kappa_i - \kappa_{\max}) \|\mathbf{\Gamma}'^{(i)}\| \, \mathrm{d}\theta \\ g_{\kappa,\mathrm{msc}}(\mathbf{c}) &= \sum_{i=1}^{N_c} \varphi\left(\frac{1}{L_i} \int_0^{2\pi} \kappa_i^2 \|\mathbf{\Gamma}'^{(i)}\| \, \mathrm{d}\theta - \kappa_{\mathrm{msc}} \right) \\ g_d(\mathbf{c}) &= \sum_{i=1}^{N_c} \sum_{j=1}^{i-1} \int_0^{2\pi} \int_0^{2\pi} \varphi(d_{\min} - \|\Gamma^{(i)}(\theta) - \Gamma^{(j)}(\theta')\|) \|\mathbf{\Gamma}'^{(i)}(\theta)\| \|\mathbf{\Gamma}'^{(j)}(\theta')\| \, \mathrm{d}\theta \, \mathrm{d}\theta' \\ g_\ell &= \mathrm{Var}\big(\{\ell_j^{(i)}\}_{j=0}^{2N_F - 1}\big). \end{aligned} \tag{5}$$

where $\mathbf{B}(\mathbf{c})$ is the field induced by the coils, L_i is the length of coil i, and κ_i is the curvature of coil i, and $\varphi(t) = \max(t, 0)^2$. In practice, we solve this problem by minimizing $f_B + w_L g_L + w_{\kappa,\max} g_{\kappa,\max} + w_{\kappa,\mathrm{msc}} g_{\kappa,\mathrm{msc}} + w_d g_d + w_\ell g_\ell$ and then increasing the weights $w_L, w_{\kappa,\max}, w_{\kappa,\mathrm{msc}}, w_d, w_\ell$, until each of the constraints is violated by at most 0.1%.

References

1. C Zhu, SR Hudson, Y Song, Y Wan, New method to design stellarator coils without the winding surface. *Nucl. Fusion* **58**, 016008 (2018).
2. F Wechsung, A Giuliani, M Landreman, A Cerfon, G Stadler, Single-stage gradient-based stellarator coil design: stochastic optimization. *arXiv:2106.12137 [physics]* (2021).
3. TG Kruger, C Zhu, A Bader, D Anderson, L Singh, Constrained stellarator coil curvature optimization with focus. *J. Plasma Phys.* **87** (2021).
